# Supplementary material for: The interactome of intact mitochondria by cross-linking mass spectrometry provides evidence for coexisting respiratory supercomplexes
Source: Mol Cell Proteomics. 2017 Dec 8;17(2):216–32. doi: 10.1074/mcp.RA117.000470 (PMC5795388; doi:10.1074/mcp.RA117.000470)
Supplement: Supplemental Data [file supp_RA117.000470_133922_0_supp_23978_fzffwf.zip › spectra_annotation/mito_DR_spectra_annotation/485-1-13-1-1-1.pdf]

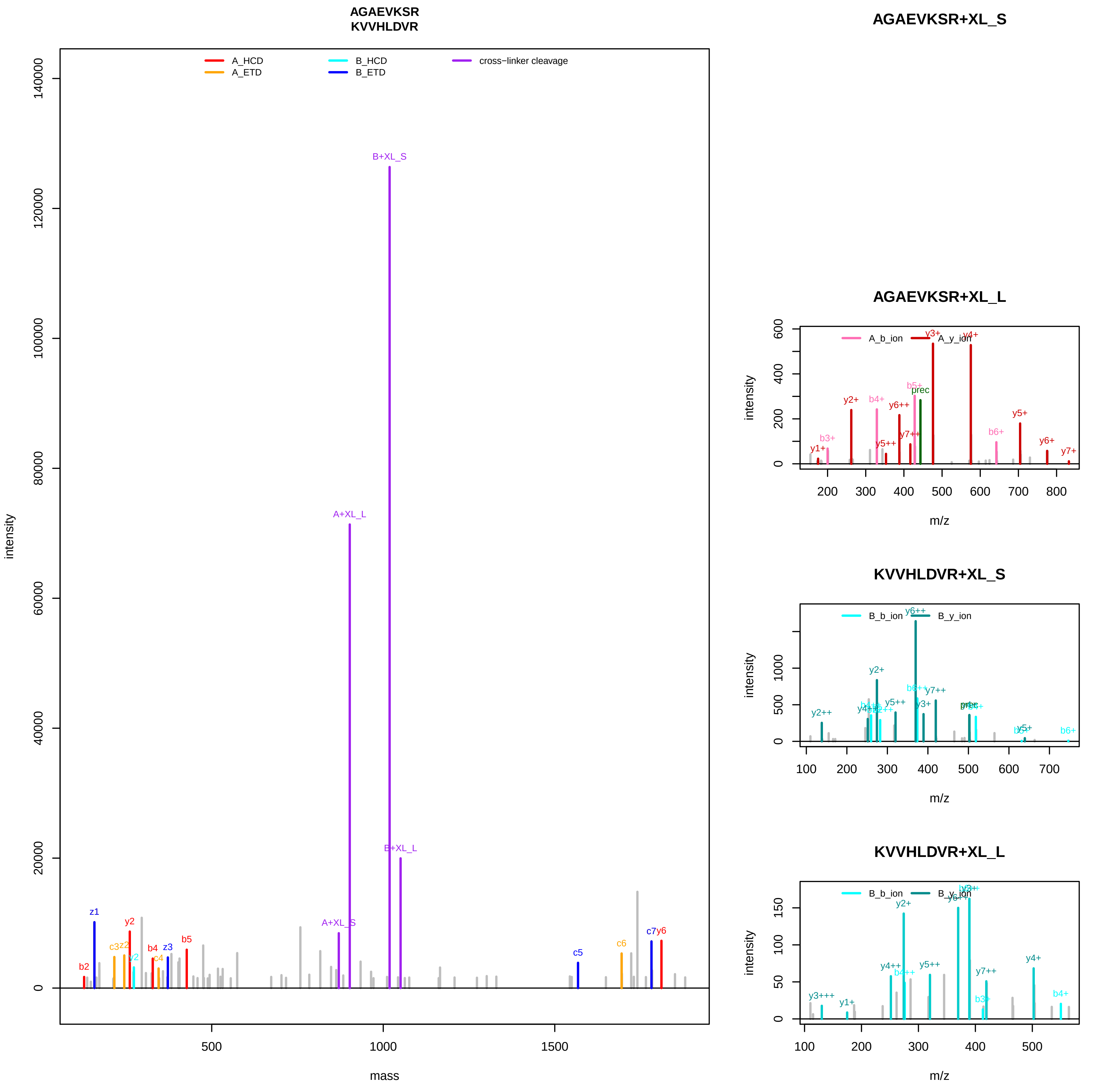

AGAEVKSR
KVVHLDVR
A_HCD
A_ETD
B_HCD
B_ETD
cross−linker cleavage
B+XL_S
A+XL_L
B+XL_L
A+XL_S
b2
z1
c3
z2
y2
b4
c4
z3
b5
c5
c6
c7
y6
intensity
mass
0
20000
40000
60000
80000
100000
120000
140000
500
1000
1500
AGAEVKSR+XL_S
AGAEVKSR+XL_L
A_b_ion
A_y_ion
y1+
b3+
y2+
b4+
y5++
y6++
y7++
b5+
prec
y3+
y4+
b6+
y5+
y6+
y7+
m/z
KVVHLDVR+XL_S
B_b_ion
B_y_ion
y6++
y2+
y2++
y5++
b6++
y7++
y3+
y5+
b6+
KVVHLDVR+XL_L
y3+++
y1+
y4++
y5++
y7++
y4+
b4+
b3+
